# The Effect of Light Intensity during Cultivation and Postharvest Storage on Mustard and Kale Microgreen Quality

**DOI:** 10.3390/antiox13091075

**Published:** 2024-09-03

**Authors:** Ieva Gudžinskaitė, Kristina Laužikė, Audrius Pukalskas, Giedrė Samuolienė

**Affiliations:** Institute of Horticulture, Lithuanian Research Centre for Agriculture and Forestry, Kaunas Str. 30, LT-54333 Babtai, Lithuania; kristina.lauzike@lammc.lt (K.L.); audrius.pukalskas@lammc.lt (A.P.); giedre.samuoliene@lammc.lt (G.S.)

**Keywords:** microgreens, kale, mustard, LED, lighting, antioxidants, shelf life, postharvest

## Abstract

Microgreens are vegetable greens that are harvested early while they are still immature and have just developed cotyledons. One of the disadvantages and a challenge in production is that they exhibit a short shelf life and may be damaged easily. In seeking to prolong the shelf life, some pre- and postharvest interventions have been investigated. Here, kale and mustard microgreens were grown in a controlled-environment walk-in chamber at +21/17 °C, with ~65% relative air humidity, while maintaining the spectral composition of deep red 61%, blue 20%, white 15%, and far red 4% (150, 200, and 250 µmol m^−2^ s^−1^ photosynthetic photon flux density (PPFD)). Both microgreens seemed to exhibit specific and species-dependent responses. Higher PPFD during growth and storage in light conditions resulted in increased contents of TPC in both microgreens on D_5_. Additionally, 150 and 250 PPFD irradiation affected the α-tocopherol content by increasing it during postharvest storage in kale. On D_0_ 150 for kale and 200 PPFD for mustard microgreens, β-carotene content increased. D_5_ for kale showed insignificant differences, while mustard responded with the highest β-carotene content, under 150 PPFD. Our findings suggest that both microgreens show beneficial outcomes when stored in light compared to dark and that mild photostress is a promising tool for nutritional value improvement and shelf-life prolongation.

## 1. Introduction

Microgreens have gained more and more interest over the past few years among consumers and researchers. Their nutritional value and benefits to human health have been amongst the main topics regarding the advantages of microgreen consumption [1]. Microgreens are early-harvested vegetable greens, harvested while they are still immature and have just developed cotyledons [2]. These immature leafy greens are considered as a superior replacement for sprouts due to their plentiful nutritional content and distinctive flavor and taste. One of the disadvantages, and a challenge in microgreen production, is that they exhibit short shelf lives and may be damaged easily [3]. In seeking to prolong the shelf life of microgreens, some pre- and postharvest interventions have been investigated [2]. One of the areas of interest is the preservation of the phytochemicals, minerals, and vitamins in which microgreens are rich [4]. With the growing demand for an uninterruptable supply of nutritious, safe, and sustainable food, controlled-environment agriculture (CEA) has attracted the attention of both science and private sectors [5]. Plants grown in controlled environments have proven to be beneficial and profitable as well as environmentally friendly [2]. Furthermore, studies have shown that modifying the lighting intensity and composition used for specific species may reduce the consumption of electricity up to 75% [6]. The modification of light parameters such as the composition, intensity, or photoperiod can also result in altered plant physiological responses and changed chemical compositions. Some studies suggest that changing the light intensity during growth may result in altered antioxidant activity during the postharvest period. For example, kale grown under a lower light intensity (30 μmol m^−2^ s^−1^) showed lower FRAP activity [7]. In other conditions, when compared, a higher light intensity resulted in higher TPC contents in kale microgreens [8]. Other studies have found that, in general, the most suitable conditions for the growth and nutritional quality of mustard microgreens included 330–440 µmol m^−2^ s^−1^ irradiation, which resulted in a higher DPPH free-radical scavenging capacity; additionally, high light levels (545 µmol m^−2^ s^−1^), which were expected to induce mild photostress, had no significant positive impact [9]. In experiments with amaranth, the results indicated that FRAP activity reached its highest values when light intensity was higher, at 280 μmol m^−2^ s^−1^ PPFD [10]. Smart light usage is important for cost reduction, and an experiment by Mlinarić et al. (2020) [11] demonstrated how the illumination of chia microgreens before harvest for 24 h caused no significant differences in total phenolics and antioxidant (DPPH) activity when compared to 48 h illumination. A preharvest treatment with light may also impact the quality of microgreens during postharvest storage, as shown via the example of mustard baby leaves, which were found to have significantly increased antioxidant capacities during storage when exposed to light at 36 μmol m^−2^ s^−1^, during growth [12]. Light may also be used during postharvest storage to change senescence dynamics. Experiments with kale [13], broccoli [14], and Brussels sprouts [15] illuminated with light during postharvest storage all showed specific and species-dependent responses to the light treatments and changes in their antioxidant activity or total phenolics. Other studies have shown even more promising results, with the potential to extend the shelf life of fresh-cut mushrooms without dramatically affecting texture and antioxidant properties identified by applying pulsed light doses during postharvest storage [16]. Although there are some studies on light’s influence on tocopherol content in mustard, beet, and parsley microgreens, which show that lower blue light doses result in higher tocopherol contents, there is a need for investigations into the influence of light intensity on microgreens’ ability to accumulate tocopherols during growth and postharvest storage. The same research suggests that higher carotenoid contents were observed while using higher doses of blue light during growth [17].

In sum, microgreens are a great source of minerals, vitamins, and other beneficial phytochemicals; a technical solution is required to prolong their shelf life and delay senescence [4]. By applying light to influence the postharvest storage of microgreens and protect their quality, there is a possibility of achieving better outcomes while using a natural and unharmful tool [2]. By finding specific growth and storage conditions for microgreens, we may contribute to better health outcomes for consumers and reduce expenses for farmers [5]. Due to the lack of sufficient research on the reaction of individual microgreens to variations in pre- and postharvest light intensity, our current study aims to provide clarity on the antioxidant responses of kale and mustard microgreens’ oxidative systems during storage. The goal was to elucidate the effects of light intensity during growth on antioxidant activity in plants during storage, as well as to compare the impacts of light exposure versus darkness during the storage period. This investigation provides valuable insights into the optimization of postharvest handling and storage conditions so as to enhance the nutritional quality and shelf life of plant-based foods, contributing to advancements in agricultural and food sciences. Furthermore, this will help in expanding our understanding of how these specific microgreens respond to light intensity changes before and after harvest, which can play a crucial role in determining their nutritional profile. By evaluating the oxidative system’s responses in these microgreens, we can shed light on their overall quality and health benefits.

## 2. Materials and Methods

### 2.1. Growth Conditions

Kale (*Brassica oleracea* L. cv ‘Red Russian’) and mustard *(Brassica juncea*. cv ‘Red lace’) microgreens were grown in a controlled environment walk-in chamber at +21/17 °C temperature and ~65% relative air humidity. Seeds for the experiments were purchased from CN Seeds (Pymoor, Ely, Cambridgeshire, UK). Seeds were spilled on a peat substrate, germinated, and grown for 10 days after germination.

**Figure 1:**
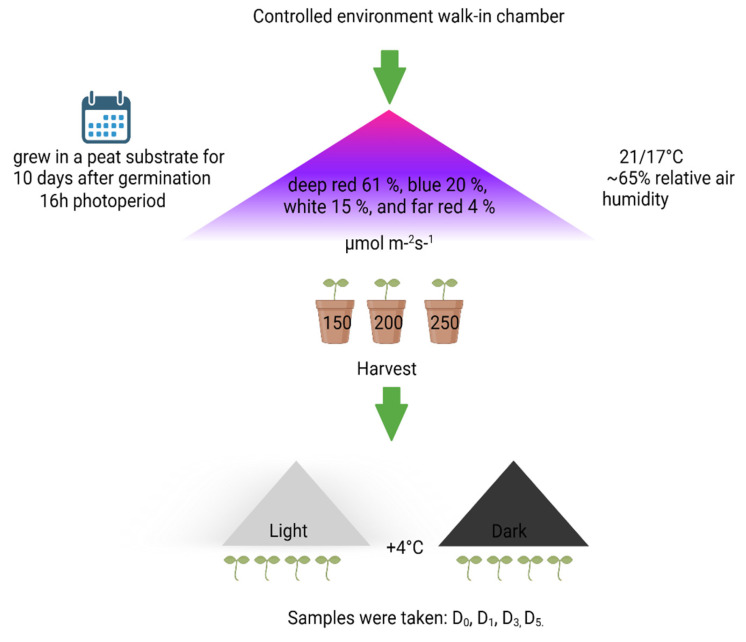
Schematic representation of microgreen cultivation conditions and sampling.

Artificial lighting was provided by 4 channel-controllable light-emitting diode (LED) lighting units (TUAS GTR 2V 0021096109 C1 DL ST, Tungsram, Budapest, Hungary), maintaining an equal spectral composition consisting of deep red 61%, blue 20%, white 15%, and far red 4% in all lighting treatments. There were three lighting intensity groups for tested microgreens, which were set at 150, 200, and 250 µmol m^−2^ s^−1^ photosynthetic photon flux density (PPFD), as graphically shown in Figure 1. PPFD was measured and regulated at the plant level using a photometer–radiometer (RF-100, Sonopan, Bialystok, Poland). After 10 days from germination, plants were harvested and stored in the dark or under white LED light (10 µmol m^−2^ s^−1^ PPFD) at +4 °C temperature in plastic containers (15 × 20 × 5 cm). Samples were collected on the harvest day (D_0_) or after one day (D_1_), three (D_3_), or five (D_5_) days of postharvest storage. Samples were frozen with liquid nitrogen and freeze-dried before performing phytochemical analysis. This experiment was carried out in a controlled-environment chamber under constant environmental conditions and replicated three times in the area; each treatment in the experimental replication consisted of three cultivation systems. Microgreens from 1.5 g of seed were cultivated in each system. All plant material from all treatment replications (*n* = 9 per treatment) was analyzed as a conjugated sample.

### 2.2. Phytochemical Analysis

Dried plant tissue was weighed on an analytical balance (Mettler Toledo AG 64, San Jose, CA, USA). Approximately 0.03 g of the plant tissue was diluted with 3 mL 80% methanol and left for 24 h in the dark at +4 °C. Then, samples were centrifuged for 10 min at 3000 rpm (Hermle Z300 K, Baden-Württemberg, Stuttgart, Germany). Sample extract was used to measure total phenolic content and antioxidant activity.

Total phenolic compounds (TPCs) were determined spectrophotometrically as described previously by Ainsworth and Gillespie [18]. Spectrophotometric analysis was performed by adding Folin–Ciocalteu reagent and 10% sodium carbonate (Na_2_CO_3_) solution to the sample extract. After 20 min, the absorbance of the mixture was measured at 765 nm, using a spectrophotometer (M501, Spectronic Camspec Ltd., Leeds, UK). The contents of TPC were quantified as gallic acid equivalents according to the calibration curve. Results were expressed as mg of TPC g^−1^ dry weight (DW).

DPPH antioxidant activity determination was performed according to the method described by Sharma and Bhat [19]. DPPH (2-diphenyl-1-picrylhydrazyl) methanol solution was added to the sample extract, incubated for 16 min, and measured at 515 nm using a spectrophotometer. The ABTS radical scavenging activity of the extracts was determined as described previously by Re et al. [20].

ABTS (2,20-azino-bis (3-ethylbenzothiazoline-6-sulphonic acid)) water solution was added to the sample extracts and they were incubated for 10–30 min and measured at 734 nm using a spectrophotometer. Both the DPPH and ABTS radical scavenging activity of extracts were expressed as Trolox equivalents (mM TE g^−1^) of dry plant weight (DW).

FRAP antioxidant activity determination was performed according to the method described by Benzie and Strain [21]. A reaction mixture consisting of 0.3 M acetate buffer, 10 mM TPTZ (2,4,6 tris(2pyridyl) s-triazine), and 20 mM FeCl was added to the sample extract, incubated for 20 min, and measured at 600 nm using a spectrophotometer. The antioxidant power was expressed as the Fe^2+^ antioxidant capacity (Fe^2+^ µM g^−1^ DW).

To identify tocopherols, extracts of the investigated microgreens were prepared by weighing 0.05 g of freeze-dried and homogenized plant material and mixing it with 5 mL of hexane (Sigma-Aldrich, St. Louis, MO, USA). Samples were placed in an ultrasonic bath (Elmasonic S 10 H, Elma Schmidbauer GmbH, Singen, Germany) for 30 min and centrifuged for 10 min at 3000 rpm (Hermle Z300 K, Baden-Württemberg, Stuttgart, Germany). The supernatant was filtered through 0.22 µm nylon syringe filters (BGB Analytic Vertrieb GmbH, Lörrach, Germany); 2 mL of supernatant was evaporated and re-diluted in 200 µL of hexane. The α-tocopherol content was evaluated by using high-performance liquid chromatography (HPLC) using a Shimadzu HPLC system (Shimadzu, Kyoto, Japan) equipped with an RF-10AXL fluorescence detector and two binary gradient pumps (LC-10AT), a vacuum degasser (DGU-14A), an autosampler (SIL-20AC), and a column oven (CTO-20AS). The column Shim-pack GIST NH2 (250 × 3 mm), particle size 5 µm (Shimadzu, Kyoto, Japan), was used for the separation of compounds. The mobile phase consisted of hexane and ethyl acetate (70:30), and separation was carried out using continuous isocratic elution at a 0.4 mL/min flow rate. A time period of 12 min was given for separation to ensure all compounds were eluted from the column. The injection volume was 3 μL and the chromatogram was monitored with a fluorescence detector set at 295 nm for excitation and 330 nm for emission wavelengths. A standard curve for each tocopherol in the range of 1.25–30 µg/mL was prepared Tocopherols were identified by comparing retention times and quantified by peak area values in comparison with corresponding standards using the linear calibration curve.

To identify and quantify carotenoids, about 0.05 g of dry ground plant material was weighed and diluted with 3 mL of 80% acetone. The extraction was carried out for 24 h at +4 °C temperature. Then, the extract was centrifuged at 10,000 rpm for 10 min and filtered through a 0.22 µm Nylon syringe filter (VWR International, Radnor, PA, USA), and HPLC analysis was performed. Contents of carotenoids were evaluated using a Shimadzu HPLC system (Shimadzu, Kyoto, Japan) consisting of DGU-14A vacuum degasser, LC-10AT HPLC pumps, an SIL-20AC autosampler, a CTO-10AS column oven, and an SPD-M10A diode array detector (DAD). Separation of compounds was performed using isocratic elution with methanol and acetone (1:1) on a YMC C30 carotenoid column (250 × 3 mm, 5 µm), at 25 °C, and the flow rate of 0.6 mL/min was used. Quantitation of compounds was performed from the DAD chromatogram, obtained at 450 and 650 nm for carotenoids and chlorophylls, respectively. The external calibration standard method was used for quantitation.

All data are presented as the mean ± standard deviation (*n* = 3 replications) and expressed on a dry weight (DW) basis. Statistical analyses were performed using MS Excel Version 2010 and the XLStat 2019 Data Analysis and Statistical Solution for Microsoft Excel (Addinsoft, Paris, France, 2019) software, using one-way ANOVA and Tukey’s HSD at the confidence level *p* ≤ 0.05. The statistical comparisons were conducted within each day’s samples to assess treatment effects, rather than across days. This approach enabled a detailed evaluation of treatment impacts within daily variations, ensuring a focused assessment of immediate treatment effects on microgreen quality. Different letters in the figures represent significant differences between treatments on each day.

## 3. Results

Antioxidant activity for both kale and mustard microgreens in all treatments on harvest day showed no significant differences in the FRAP, ABTS and DPPH assays. Similarly, TPC contents showed insignificant differences between treatments on days D_0_ and D_1_ for both light and dark storage conditions.

**Figure 2:**
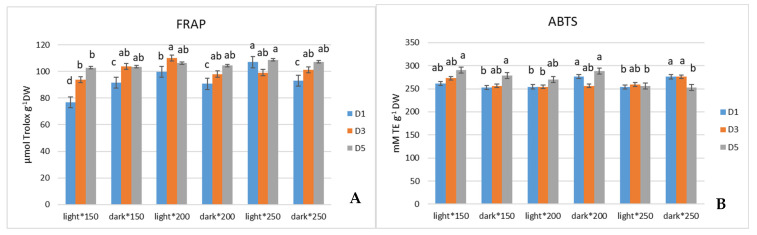

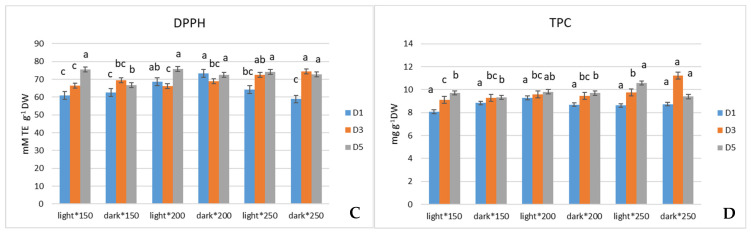
Effect of light intensity during cultivation on FRAP (**A**), ABTS (**B**), and DPPH (**C**) activity and TPC content (**D**) of kale (*Brassica oleracea*) extracts during postharvest storage (*p* < 0.05).

On D_3_ of storage, the highest TPC was found in microgreens grown under 250 µmol m^−2^ s^−1^ and stored in the dark, although on D_5_, the highest TPC was found in kale grown under 250 µmol m^−2^ s^−1^ but stored in the light (Figure 2D).

When evaluated by the FRAP assay, kale microgreens showed a tendency to exhibit the highest antioxidant activity when grown under 250 µmol m^−2^ s^−1^ PPFD and stored in the light. This tendency seemed to be evident until the last D_5_ of storage, and on D_5_, samples had the same activity as on D_1_ (Figure 2A). ABTS assay shows, on D_1_, the highest activity to be in microgreens grown under 200 and 250 µmol m^−2^ s^−1^ PPFD stored in the dark, but during storage on D_5_, antioxidant activity of 250 µmol m^−2^ s^−1^ seemed to have the most dramatic decrease, while 200 µmol m^−2^ s^−1^ seemed to maintain the highest antioxidant activity up until D_5_ (Figure 2B). The DPPH assay showed the highest values on D_1_ to be in kale microgreens grown under 200 µmol m^−2^ s^−1^ independent of whether they were stored in light or dark conditions. Although on D_5_ of postharvest storage, the significant difference seemed to be only in kale grown under 150 µmol m^−2^ s^−1^, which proved to have the lowest antioxidant activity (Figure 2C).

**Figure 3:**
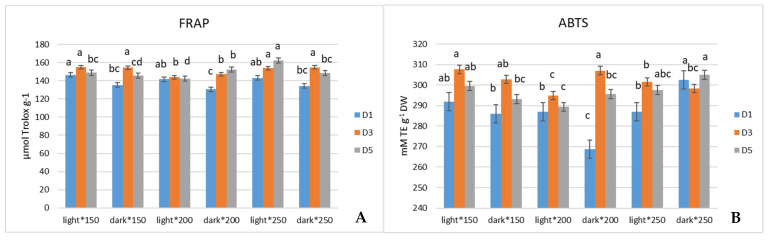

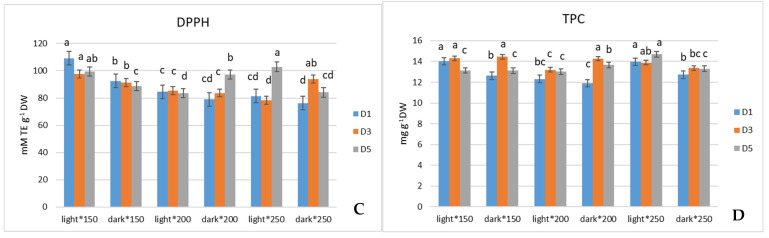
Effect of 150, 200, and 250 µmol m^−2^ s^−1^ PPFD LED treatment on FRAP (**A**), ABTS (**B**), and DPPH (**C**) activity and TPC (**D**) of mustard (*Brassica juncea)* during postharvest storage (*p* < 0.05).

Antioxidant properties of the analyzed mustard microgreens evaluated by the FRAP assay, on D_1_ of postharvest storage, showed higher results when stored in the light compared to those stored in the dark in all treatments. On D_3_, it became apparent that the significantly lowest activity was found in mustard grown under 200 µmol m^−2^ s^−1^, independently of whether it was stored in the light or dark. Although, on D_5_, 200 µmol m^−2^ s^−1^ treatment showed separation between light and dark storage conditions. Mustard stored in the light had significantly lower activity than that stored in the dark on D_5_ (Figure 3A). Contrary to the FRAP assay results, the ABTS assay showed that, on D_1_, the highest activity was in microgreens grown under 200 µmol m^−2^ s^−1^ and stored in the dark (Figure 3B). DPPH antioxidant activity on D_1_ and D_3_ was determined to be highest in mustard microgreens grown under 150 µmol m^−2^ s^−1^ and stored in the light. On D_5_,the highest value was found to be in microgreens with the most extreme growth conditions of −150 and 250 µmol m^−2^ s^−1^, both stored in the light (Figure 3C).

Similarly to the DPPH antioxidant activity response on D_5_, the TPC contents showed the highest amounts while grown under the most extreme conditions used. Microgreens grown under 150 and 250 µmol m^−2^ s^−1^ and stored in the light contained higher TPC contents after one day of postharvest storage, and on D_3_, plants that received 200 µmol m^−2^ s^−1^ PPFD treatment seemed to exhibit the lowest contents of TPC. On D_5_ of storage, the significance in differences between TPC contents in mustard microgreens seemed to be lost, and only 250 µmol m^−2^ s^−1^ PPFD treatment mustard had significantly higher TPC contents when stored in the light (Figure 3D).

**Figure 4:**
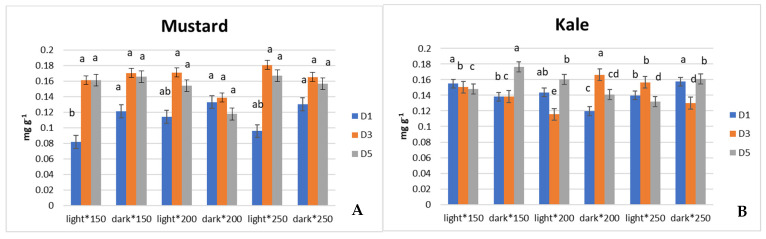
Effect of light intensity during growth on α-tocopherols of mustard (*Brassica oleracea*) (**A**) and kale (*Brassica juncea)* (**B**) during postharvest storage (*p* < 0.05).

On harvest day (D_0_), α-tocopherol contents showed no significant differences between the treatments for kale and mustard microgreens. On D_1_ of postharvest storage, for mustard microgreens, significantly higher contents of α-tocopherol were determined in plants stored in the dark than in the light (Figure 4A). On the contrary, for kale microgreens, on D_1_ of storage, 200 µmol m^−2^ s^−1^ PPFD treatment when stored in the dark resulted in the lowest α-tocopherol contents. On D_3_, the light storage conditions seemed to produce a decrease in α-tocopherol contents in the same mentioned 200 µmol m^−2^ s^−1^ PPFD in kale microgreens. On D_5_, lower levels of tocopherols appeared in kale grown under 200 and 250 µmol m^−2^ s^−1^, while microgreens grown under 150 µmol m^−2^ s^−1^ showed significantly increased levels compared to all the other treatments (Figure 4B). On storage days D_3_ and D_5_, the differences between treatments were insignificant for mustard microgreens.

**Figure 5:**
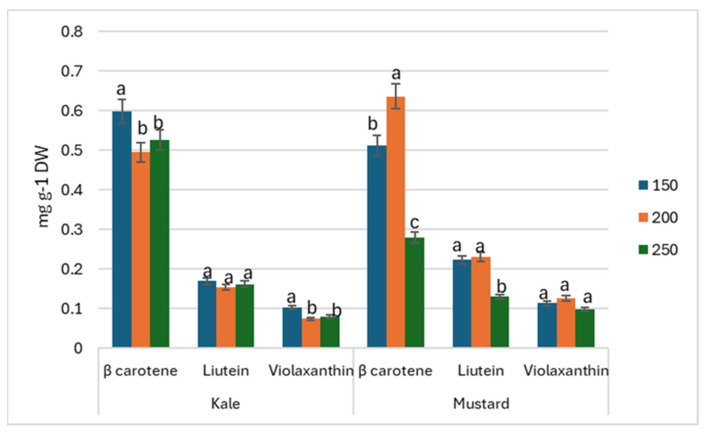
Effect of 150, 200, and 250 µmol m^−2^ s^−1^ PPFD LED treatment on carotenoids of kale (*Brassica oleracea)* and mustard (*Brassica juncea)* during harvest (*p* < 0.05).

The determined β-carotene contents showed a common tendency for increased PPFD to result in decreased accumulation of β-carotene, lutein, and violaxanthin on harvest day (Figure 5). Significant differences were detected on harvest day (D_0_) for both kale and mustard microgreens. On D_0_, kale accumulated the highest amounts of β-carotene and violaxanthin while grown under 150 µmol m^−2^ s^−1^ PPFD treatment, while mustard exhibited higher amounts when grown under 200 µmol m^−2^ s^−1^ PPFD (Figure 5). Violaxanthin was on the same level in all PPFD doses.

**Figure 6:**
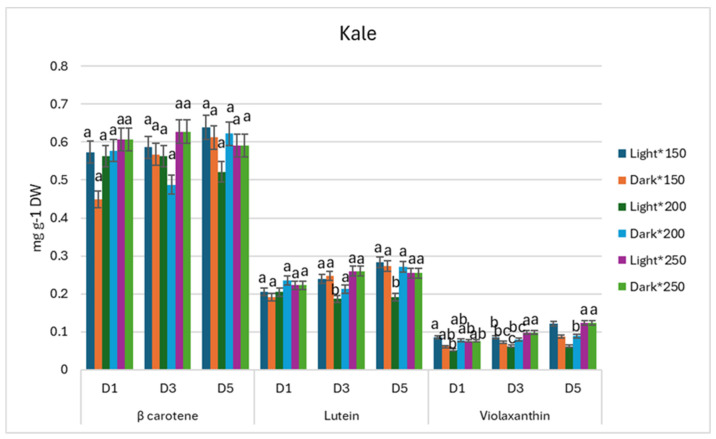
Effect of 150, 200, and 250 µmol m^−2^ s^−1^ PPFD LED treatment on carotenoids of *Brassica oleracea* during postharvest storage (D_1_,D_3_,D_5_) (*p* < 0.05).

During storage, β-carotene contents showed no significant differences between treatments in kale microgreens, while the significantly lowest contents of lutein were obtained in kale microgreens grown under 200 µmol m^−2^ s^−1^ PPFD and stored in the light. The PPFD of 200 µmol m^−2^ s^−1^ during growth and postharvest storage in light seemed to maintain a tendency to significantly decrease violaxanthin contents during postharvest storage on D_3_ and D_5_ (Figure 6).

**Figure 7:**
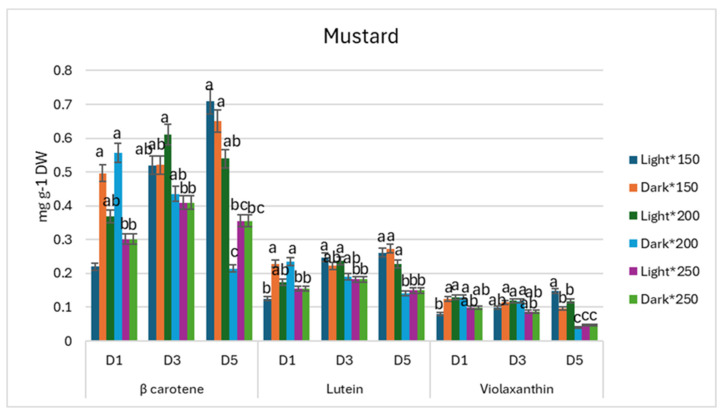
Effect of 150, 200, and 250 µmol m^−2^ s^−1^ PPFD LED treatment on carotenoids of mustard (*Brassica juncea*) during postharvest storage (D_1_, D_3_, D_5_) (*p* < 0.05).

On the other hand, the accumulation of carotenoids in mustard microgreens showed different tendencies. The lowest β-carotene, lutein, and violaxanthin contents we found on D_1_ when plants were grown under (the lowest) 150 µmol m^−2^ s^−1^ PPFD and stored in light, and during postharvest on D_5_, the β-carotene, lutein, and violaxanthin contents reached the highest amounts under lower-intensity treatments, which suggests carotenoid retention in the microgreens (Figure 7).

## 4. Discussion

The novelty of this research lies in the comprehensive approach to optimizing microgreen cultivation and postharvest quality through controlled-environment agriculture. Utilizing a controlled-environment chamber ensures precise and replicable growing conditions, isolating the impacts of different LED light intensities on microgreens. This focus on LED lighting, a cutting-edge technology, allows for the exploration of specific wavelengths and intensities, providing valuable insights into their effects on plant growth and development. Extending beyond the growth phase, this work uniquely examines the postharvest storage quality, specifically antioxidant activity, highlighting how pre-harvest conditions influence shelf life and nutritional value. This integrated approach not only advances scientific understanding but also offers practical applications for urban farming, vertical farming, and other controlled-environment agricultural practices, ultimately contributing to the production of healthier, longer-lasting microgreens [5].

In this study, both kale and mustard microgreens seemed to exhibit specific and species-dependent responses. Likewise, PPFD intensity treatments led to insignificant TPC and antioxidant activity differences in both microgreens on D_0_. In agreement with Hernández-Adasme et al. [22], beet microgreens were grown under 120, 160, and 220 µmol m^−2^ s^−1^ and no significant differences in antioxidant activity were determined, although some studies suggest that 210 μmol m^−2^ s^−1^ PPFD increases TPC contents in *Brassicaceae* species [23]. In other experiments with kale microgreens, when the influence of light intensity was evaluated (30, 50, 70, and 90 µmol m^−2^ s^−1^), the results showed no significance when the DPPH activity response was evaluated, but the FRAP activity response was significantly lower in microgreens grown under 30 µmol m^−2^ s^−1^ compared to other treatments [7]. In general, the most suitable condition for mustard growth and nutritional quality was 330–440 µmol m^−2^ s^−1^ light intensity, which resulted in a higher DPPH free-radical scavenging capacity, while a high light level (545 µmol m^−2^ s^−1^), which was expected to induce mild photostress, had no significant positive impact [9]. A high irradiation level (280 μmol m^−2^ s^−1^ PPFD) showed a FRAP activity response in terms of an increase in activity in amaranth [10]. Although contrary to our findings, the literature states that a 150 µmol m^−2^ s^−1^ lighting intensity increases TPC contents when compared to 50 and 100 µmol m^−2^ s^−1^ [8]. Our findings suggest that both mustard and kale microgreens show beneficial outcomes when stored in light, compared to dark storage, in regard to changes in DPPH assay. In agreement, Brussels sprouts’ illumination with 20 µmol m^−2^ s^−1^ PPFD during storage showed an increase in DPPH and ABTS activity in comparison to those stored in the dark [15].

Similarly, higher PPFD during growth resulted in increased contents of TPC in both microgreens, and by the last day of storage, both mustard and kale seemed to show the highest antioxidant response when grown under 250 µmol m^−2^ s^−1^ PPFD and stored in the light. Green and red kale illumination during storage also results in increased TPC contents when compared to the dark treatment [13]. Broccoli irradiation with 12 µmol m^−2^ s^−1^ PPFD during postharvest storage suggests a significant response in terms of TPC contents’ increase, when compared with controls, along with senescence reduction [14]. Chia microgreen’s reaction to light exposure (100 µmol m^−2^ s^−1^) for different periods of time before harvest showed the content of total phenolics and antioxidant (DPPH) activity to increase, but no distinctive difference was found between illumination times [11]. Mustard baby leaves showed exposure to light (36 μmol m^−2^ s^−1^) during postharvest storage to significantly increase antioxidant capacity and prolong storage quality [12], while other studies showed even more promising results, with a potential to extend the shelf life of fresh-cut mushrooms without dramatically affecting texture and antioxidant properties by applying pulsed-light doses during postharvest storage [16]. Moreover, 150 and 250 PPFD irradiation increased the α-tocopherol content during postharvest storage in kale microgreens. Even though there were no significant differences in α-tocopherol contents between plants stored in the dark or light, 200 μmol m^−2^ s^−1^ PPFD growing conditions clearly showed α-tocopherol contents to decrease. Although higher light doses on mustard, beet, and parsley microgreens led to increase tocopherol contents [17], there is a need for an investigation of light intensity’s influence on microgreens’ ability to accumulate tocopherols during growth and postharvest storage.

β-carotene responses to light treatments were species dependent. On D_0_, 150 μmol m^−2^ s^−1^ for kale and 200 μmol m^−2^ s^−1^ PPFD for mustard showed microgreens to respond in terms of β-carotene accumulation. On D_5_, kale seemed to show insignificant differences between treatments, while mustard microgreens responded with the highest β-carotene contents while under 150 μmol m^−2^ s^−1^ PPFD. The same response to accumulate higher amounts of carotenoids was obtained with mustard under lower (220 μmol m^−2^ s^−1^) irradiation; however, red pak choi and tatsoi show a propensity to accumulate higher amounts of carotenoids while grown under greater illumination (440 μmol m^−2^ s^−1^) [24]. Amaranth microgreens, meanwhile, seem to respond with an insignificant impact on carotenoid contents under 130–280 μmol m^−2^ s^−1^ [10], although research with broccoli microgreens showed a decrease in carotenoid contents while increasing the light intensity (from 30 to 90 μmol m^−2^ s^−1^) and indicated 70 μmol m^−2^ s^−1^ to be the most beneficial for the accumulation of phytochemicals [25].

During storage, PPFD irradiation treatments showed no impact on β-carotene contents in kale microgreens, while the lowest lutein accumulation occurred under 200 µmol m^−2^ s^−1^ PPFD treatment and while stored in the light. The irradiation of 200 µmol m^−2^ s^−1^ PPFD during growth resulted in a decrease in antioxidant compound accumulation, such as violaxanthin, during storage. In agreement with our study, radish microgreens’ storage in the dark resulted in carotenoid retention [26]. On the other hand, mustard microgreens showed different tendencies. All the lowest β-carotene, lutein, and violaxanthin accumulation on D_1_ were seen in 150 µmol m^−2^ s^−1^ PPFD while stored in the light, but during postharvest, on D_5_, β-carotene, lutein, and violaxanthin contents reached the highest amounts when compared with the other treatments, which suggests carotenoid retention in the microgreens. In agreement with our findings, the literature discloses that light exposure during storage can result in enhanced carotenoid contents for mustard baby leaves [12].

Even though specific antioxidants’ responses to light treatments are hard to predict, plants primarily deal with stress through two main mechanisms consisting of different enzymatic (SOD, CAT, APX, GR, MDHAR, DHARl, etc.) and nonenzymatic (ascorbic acid, glutathione, phenolic acids, alkaloids, flavonoids, carotenoids, α-tocopherol, nonprotein amino acids, etc.) antioxidants [27]. Maintaining an optimum level of stress is important for plant acclimation and, as some of the literature states, in cellular proliferation and differentiation, which may lead to an increased yield or nutritional value [28]. In agreement, our research suggests mild photostress to be a promising tool for nutritional value improvement and shelf-life prolongation for mustard and kale microgreens. Furthermore, it shows a species-specific response to light exposure, which leads us to call for further investigations of the specific antioxidant response to PPFD irradiation during cultivation and in postharvest storage.

## Data Availability

Data is contained within the article.
